# La place du clou Telegraph court dans le traitement des fractures de l'extrémité supérieure de l'humérus: à propos de 19 cas

**DOI:** 10.11604/pamj.2016.24.36.8709

**Published:** 2016-05-10

**Authors:** Mohamed Amine karabila, Ismail Hmouri, Younes Mhamdi, Mohamed Azouz, Tarik Madani, Mohamed Kharmaz, Mohamed Ouadaghiri, Moulay Omar Lamrani, Ahmed Bardouni, Abdou Lahlou, Mustapha Mahfoud, Mohamed Saleh Berrada, Éric Montbarbon, Emmanuel Beaudouin

**Affiliations:** 1 Service de Chirurgie Orthopédique et de Traumatologie, CHU Ibn Sina, Rabat, Maroc; 2 Service de Chirurgie Orthopédique et de Traumatologie, Centre hospitalier de Chambéry, France

**Keywords:** Clou, humérus, fracture, Nail, humerus, fracture

## Abstract

La fracture de l'extrémité supérieure de l'humérus est la troisième fracture en fréquence chez les sujets âgés et leur répartition est bimodale touchant préférentiellement le sujet âgé ostéoporotique après un traumatisme à faible énergie ou plus rarement le sujet jeune par mécanisme à forte cinétique. Le traitement des fractures complexes de l'humérus proximal est le sujet de nombreuses controverses Le clou Telegraph constitue une approche thérapeutique très efficace pour les fractures déplacées de l'extrémité supérieure de l'humérus, de technique chirurgicale facile mais avec une courbe d'apprentissage et permettant un protocole de rééducation dans l'immédiat de l'intervention. C'est un matériel d'enclouage antérograde de 15 cm de long, plein verrouillé en proximal et en distal, le verrouillage proximal est assuré par 4 vis spongieuses, de filetage long, stables dans le clou et cela confère une solidité tout-à-fait remarquable à ce montage alors que le verrouillage distal est assuré au niveau du V deltoïdien en zone avasculaire et là où il n'y a pas de passage nerveux. L’étude présentée concerne 19 patients traités par un clou Telegraph court dans le traitement des fractures de l'extrémité supérieure de l'humérus entre 2013 et 2015 et elle a pour but d'analyser les résultats radio-cliniques et d’évaluer la répercussion de cette technique sur la fonction de l’épaule. Le clou Telegraph proposé depuis plus de 12 ans à peu près, a rencontré et continue de rencontrer un réel succès. Il permet de traiter très efficacement les fractures simples type 2 et 3, mais aussi les fractures impactées en valgus à 4 fragments. L'ostéosynthèse par clou Telegraph est une solution efficace, rapide et reproductible dans le traitement chirurgical des fractures de l'extrémité supérieur de l'humérus même en cas des fractures complexes et permet une reprise rapide de la mobilité de l’épaule.

## Abstract (en)

The fracture of the upper end of the humerus is the third most frequent fracture in the elderly and its distribution is bimodal, preferentially affecting osteoporotic elderly patients after low energy trauma or, more rarely, young subjects as a consequence of severe kinetic mechanism The treatment of complex fractures of the proximal humerus is the subject of much controversy. Telegraph nail is a highly effective therapeutic approach for displaced fractures of the upper end of the humerus. Surgical procedure is easy but with a learning curve; it allows to start rehabilitation protocol immediately after surgery. It is an antegrade nailing material, 15 cm long, with full proximal and distal locking. Proximal locking is secured by 4 cancellous screws, long thread, stable in the nail ensuring perfect solidity of the assembly while the distal locking screw is secured to deltoid tuberosity in the avascular zone and where there is no neural pathway. This study reports 19 case of patients with fractures of the humerus treated with short Telegraph nail between 2013 and 2015. It aims to investigate radio-clinical results and to assess the impact of this technique on shoulder function. Telegraph nail that has been used for more than 12 years or so met and continues to meet with strong success. It allows to trat very effectively simple fractures type 2 and 3, but also 4-fragment valgus impacted fractures Osteosynthesis by Telegraph nail is an efficient, rapid and reproducible surgical treatment option of the fractures of the upper end of the humerus even in case of complex fractures and allows for a more rapid return of shoulder mobility.

## Introduction

Les fractures de l´extrémité supérieure de l´humérus représentent environ 4% de la totalité des fractures. Leur fréquence passe à plus de 10% au-delà de 65 ans, leur incidence est en constante augmentation avec le vieillissement de la population. 80% à 85% des fractures de l'extrémité supérieure de l'humérus sone peu ou pas déplacées et peuvent bénéficier d'un traitement orthopédique avec des bons résultats [1–4]. Cette notion d'absence de déplacement reste purement arbitraire: Neer la définie comme une bascule de la tête inférieure à 45° ou/et un déplacement des fragments de moins de 1 cm. Environ 20% de ces fractures, instables ou à grand déplacement nécessitent une prise en charge chirurgicale [5]. Les fractures proximales de l´humérus sont caractérisées par les difficultés de leur traitement, l´absence de technique de référence et certaines controverses sur les indications chirurgicales et les techniques opératoires. Les résultats sont remarquables pour les fractures articulaires impactées et les fractures extra-articulaires alors que les fractures articulaires complexes (type 4 de Neer) fortement déplacées s'accompagnent des résultats décevants et relèvent d'un traitement prothétique hormis chez les sujets jeunes [6–8].

## Méthodes

**Figure 1:**
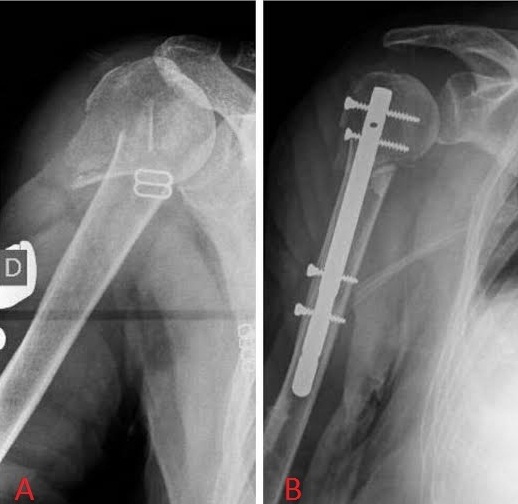
A) fracture type 2 de Neer déplacée; B) contrôle post-opératoire avec bonne reduction

**Figure 2:**
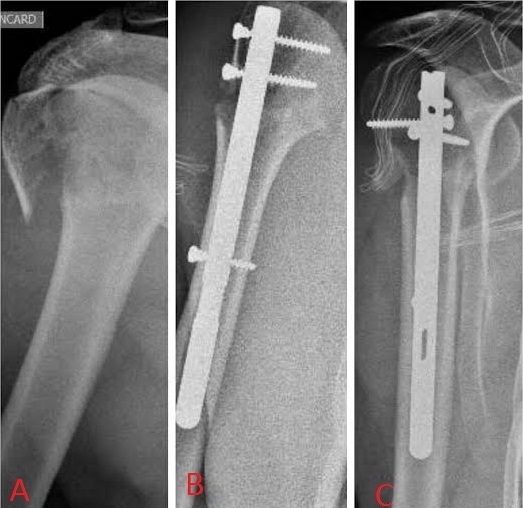
A) fracture type 3 (col chirurgical et trochiter) de Neer; B et C) bon contrôle radiologique

**Figure 3:**
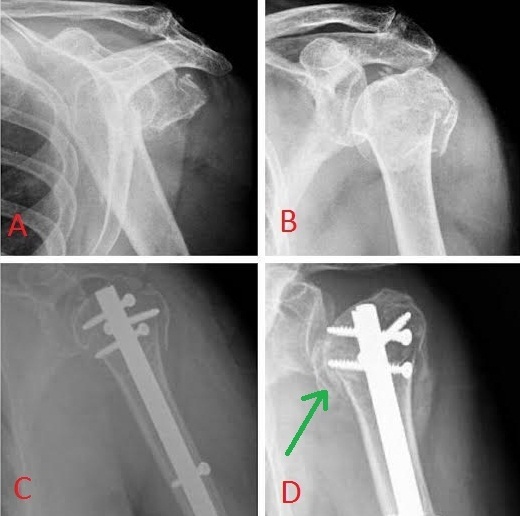
A et B) fracture complexe impactée en valgus type 4 de Neer cher un sujet âgé; C et D) bonne réduction et consolidation de la fracture mais début de l'ostéonécrose de la tête

**Figure 4:**
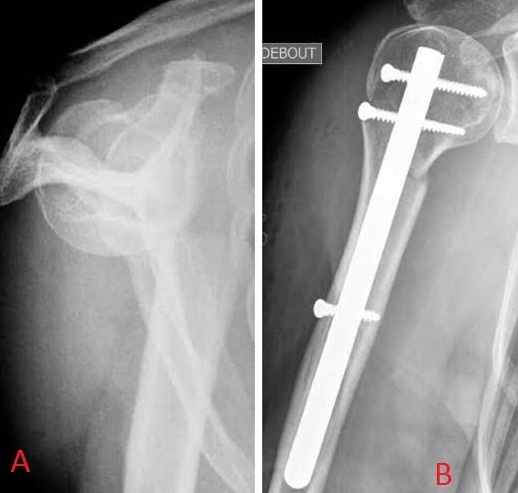
A) fracture-luxation de la tête humérale; B) réduction par manœuvre externe et clou Telegraph en percutané avec bon contrôle post-opératoire

Il s'agit d'une série prospective de 19 patients étalée entre 2013 et 2015 traités par clou Telegraph, il y avait 12 femmes (63%) pour 7 hommes (37%) avec un recul moyen de 11 mois, la moyenne d’âge était de 62 ans. Un abord classique antéro-externe a été utilisé dans tous les cas en position semi-assise. Les fractures survenues sur un os pathologiques les traitements orthopédiques et les pseudarthroses initiales ont été exclus de cette étude et la fracture a été évaluée en fonction de la classification de Neer. Nous avons étudiés les résultats chez les patients revus à au moins 6 mois post-opératoire par un examen clinique et radiographique pour évaluer la fonction de l’épaule, juger la consolidation et rechercher une éventuelle complication. Nombreuses classifications ont été proposées, mais la plus répandue et utilisée chez nous est celle de Neer qui divise l'extrémité supérieure de l'humérus en 4 fragments. 9 patients présentaient une fracture du col chirurgical à deux fragments (Figure 1) selon la classification de Neer, 7 cas avec une fracture à trois fragments (Figure 2), un cas avec une fracture à quatre fragments (Figure 3) et un cas avec une fracture-luxation (Figure 4). 16 cas parmi nos patients étaient suite à une chute mécanique simple, les autres suites à des accidents de la voie publique et il y avait un cas de fracture ouverte. L'indication opératoire prenait comme critère une fracture avec un diastasis inter-fragmentaire de plus d'un centimètre et une bascule de plus de 45° de la surface articulaire humérale (par rapport aux 130° de référence). Tous nos patients étaient traités par un abord atéro-latéral en percutané et nous avons optés toujours pour un verrouillage distal dynamique. L’épaule était immobilisée pendant 3 semaines en moyenne et la mobilisation postopératoire pendulaire était à partir du troisième jour. Les résultats fonctionnels sont appréciés par le score de Constant.

## Résultats

**Patient:** La moyenne d’âge était de 62 ans. Il n’était pas retrouvé de différence d’âge en fonction du sexe. En moyenne, l'intervention s'est faite à 4 jours (1 à 15 jours) du traumatisme.

**Consolidation:** Toutes les fractures ont consolidé avec un délai moyen allant de 8 à 12 semaines. La consolidation a été acquise en position acceptable dans tous les cas. Nous n'avons pas relevés des cas de pseudarthrose.

**Fonction de l’épaule:** Au dernier recul les mobilités postopératoires de l’épaule, la flexion moyenne était de 130° avec des extrêmes de 80° et 160° et l'abduction de 120° avec des extrêmes de 90° et 150°. En comparant les scores de Constant, 9 résultats sont évalués comme excellents, 7 sont bons et 3 moyens (Tableau 1). Le score de constant moyen était de 85,7 points/100 avec un minimum de 49.

**Tableau 1:** Résultats de score de Constant pondéré

Score de constant en %	Nombre de patients
>90	9
80-89	7
<80	3

**Figure 5:**
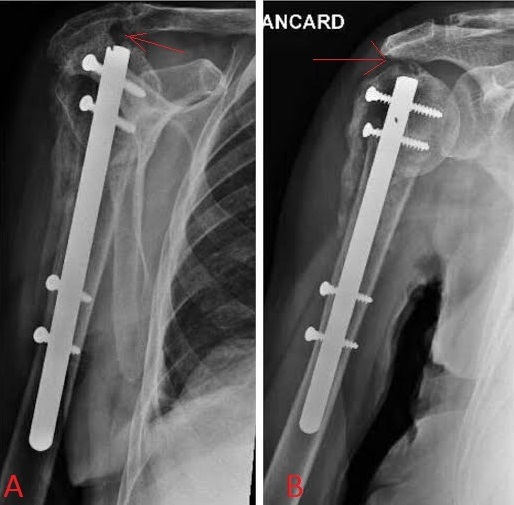
A et B) conflit sous acromial

**Complications:** Nous n'avons pas eu de complications immédiates vasculaire ou nerveuse Parmi ces 19 patients, nous déplorons un cas d'algoneurodystrophie (5%), une rupture d'une vis proximale a été notée chez un patient (5%) et nous avons retrouvé un cas de conflit sous acromial (5%) chez un seul patient (Figure 5). Un cas de nécrose partielle (5%) de la tête de l'humérus a été noté après 8 mois d’évolution (Figure 3).

**Résultats radiologiques:** Selon les critères de réduction, les résultats se répartissent en 9 réductions anatomiques, 4 sub-anatomiques, 4 intermédiaires et 2 mauvais. Nous n'avons pas notés de déplacements secondaires manifestes (le déplacement secondaire est défini par une valeur à 10° pour définir une variation de la bascule céphalique et 10% pour une modification, de la translation de face et de profil).

## Discussion

Les fractures de l'extrémité supérieure de l'humérus sont très fréquentes, de pronostic et de traitement très différents. Elles se positionnent au troisième rang des fractures des sujets de plus de 65 ans [9], derrière les fractures du poignet et du fémur, avec une nette prédominance féminine [10]. Vue l'augmentation de l'espérance de vie et du vieillissement de la population, la fréquence de ces lésions post-traumatiques est en augmentation progressive et durant ces dernières années, leur taux a été multiplié par trois au-delà de 60 ans [11] et selon de récentes projections toutes les femmes âgées actuellement de 65 ans présenteront un jour une fracture de l'extrémité proximale de l'humérus [12]. La prise en charge de ces fractures peut varier du simple traitement orthopédique immobilisation de l’épaule à l'arthroplastie humérale, en passant par de nombreuses techniques d'ostéosynthèse. Ces lésions post-traumatiques posent trois problèmes pour le chirurgien: le premier problème est défi diagnostique qui nécessite l'identification des traits, des déplacements et une bonne classification de la fracture en se basant sur des images radiographiques de bonne qualité et des incidences radiologiques bien posées type radiographie de l’épaule de face classique oblique à 20°, profil de Lamy ou de Neer et profil de coiffe et dans les fractures complexe ou comminutives, l’étude est simplifiée par le scanner de l’épaule avec reconstruction de la fracture en 3D. Le second problème est thérapeutique, les fractures peu ou pas déplacées ne nécessitent qu'un traitement orthopédique alors que les fractures déplacées nécessitent un traitement chirurgical. Le troisième problème est pronostique car ces fractures peuvent compromettre, altérer voir engager le pronostic fonctionnel de l’épaule par le biais de douleurs résiduelles, d'une raideur et d'une diminution de la force. Le pronostic des fractures extra-articulaires est néanmoins plus favorable que celui des fractures articulaires complexes, menacées d'arthrose et de nécrose céphalique. Le clou Telegraph proposé depuis plus de 12 ans à peu près, a rencontré et continue de rencontrer un réel succès. Aujourd´hui, il semble que c´est la meilleure solution et qu´il n´a pas d´équivalent. Il permet de traiter très efficacement [13] les fractures simples c´est-à-dire les fractures à 2 et 3 fragments de Neer, mais aussi les fractures impactées en valgus à 4 fragments. Le traitement chirurgical des fractures de l'extrémité proximale de l'humérus par clou Telegraph est une technique très fiable favorable. Le clou présente une résistance mécanique globale similaire et satisfaisante vis-à-vis d'une pression axiale et semblait être plus adaptée mécaniquement et permet une mobilisation précoce de l’épaule, elle s'opposait au mieux à la latéralisation de la tête et à l’écartement des tubérosités. Ses propriétés biomécaniques semblent même supérieures aux dispositifs d'ostéosynthèse extra-osseux: le clou est plus rigide par rapport aux plaques vissées et assure donc une plus grande stabilité primaire aussi bien dans la région diaphysaire que métaphysaire. Il semble également avoir des qualités biomécaniques supérieures pour des contraintes de type torsion et cintrage permettant la possibilité de démarrer le protocole de rééducation le plus rapidement possible.

Généralement les fractures type 2 de Neer ne posent pas des problèmes de réduction une fois la bascule ou la translation de la tête humérale est corrigée, par contre les fractures type 3 ou 4 de Neer peuvent poser de réduction de la tête et des tubérosités et c'est la d'où vient l'intérêt du clou avec ses 4 vis proximales poly-axiales (frontale, sagittale et oblique) autorisant une meilleure fixation du trochiter et du trochin. La réduction du fragment trochitérien est indispensable et connue de longue date [14–16]. Sous l'action des muscles supra et infra épineux, le trochiter se déplace toujours en postérieur et en proximal, ainsi une consolidation en position ascensionnée entraîne un conflit sous acromial [17] et en position postérieure un conflit avec la glène [18]. Les lésions de la coiffe des rotateurs secondaires à la saillie du clou, ainsi que celles qui sont engendrées lors de l'ostéosynthèse initiale puis à l'ablation du matériel est un des arguments majeurs des détracteurs de l'enclouage antérograde. Boughebri [19] publie en 2007 une étude prospective qui conclut que la qualité de la réduction de la fracture était un paramètre important influant sur le résultat clinique final, avec un score de Constant moyen de 88,7% pour les fractures dont la réduction était jugée correcte, contre 75,6% lorsqu'elle était mauvaise. Les complications retrouvées dans notre série sont en accord avec celles qui sont retrouvées dans la littérature. La série réalisée par Cuny publiée en 2002 [20] présente 15 conflits secondaires à un clou saillant ou à une mobilisation d'une vis de verrouillage (26%) alors que dans notre série le taux est nettement diminué (5%). Trois reprises chirurgicales (5,3%) ont été nécessaires par insuffisance de réduction lors de la première intervention. Une ostéonécrose (1,75%) a été retrouvée pour 64 fractures et pour nous nous avons constaté une ostéonécrose pour 19 patients sur une fracture complexe type 4 de Neer ou un taux de 5%. En 2003 et 2008 [21, 22] à propos des fractures type 3 et 4, il retrouve la même proportion de survenue de saillie de clou. Comme s’était montré dans l'enclouage centromédullaire qui avait un faible risque d'infection, nous n'en constatons aucune complication septique profonde. L'ostéonécrose de la tête c´est une complication fréquemment retrouvée après les fractures de l´humérus proximal. Les statistiques varient entre 15 et 50%.comme Gerber [23] a souligné ces nécroses étaient souvent bien supportées par les patients: élévation à 80, 90° et pas de douleurs comme dans notre cas: abduction 70° et flexion 80°. Pour l´auteur, les facteurs de mauvais résultats conduisant à une nécrose sont une ostéosynthèse imparfaite ou des lésions concomitantes des tendons de la coiffe des rotateurs. Notre étude peut être soumise à plusieurs critiques. D'abord, il s'agit d'une étude observationnelle descriptive prospective. En outre, la taille de notre série est assez réduite et un biais majeur est la participation à cette étude de plusieurs chirurgiens d'expérience très variables.

## Conclusion

L'ostéosynthèse des fractures de l'humérus proximal par clou Telegraph nous apparaît comme solution valable et permet d'obtenir de bons résultats radio-cliniques. La technique nécessite une réalisation rigoureuse et une bonne réduction initiale de la translation et du fragment trochitérien grâce à l'installation du patient et les manœuvres réductionnels externes avant l'ostéosynthèse et nous recommandons donc l´utilisation de ce clou dans un premier temps pour les fractures simples à deux et trois fragments ainsi que, mais à un degré moindre car c´est quelquefois difficile, pour les 4 fragments impactées en valgus pour les sujets jeunes qui offrent une grande flexibilité pour obtenir une réduction acceptable.

### Etat des connaissances actuelle sur le sujet

- Pathologie de plus en plus fréquente du fait de vieillissement de la population et de l'ostéoporose.
- Troisième fracture en fréquence chez les sujets âgés.
- Le diagnostic clinique et radiologique et bien codifiée par les auteurs et la littérature mais la démarche thérapeutique à adopter reste souvent intuitive basée sur l'expérience du chirurgien et sur le type de fracture.

### Contribution de notre étude à la connaissance

- L'ostéosynthèse par clou Telegraph nous parait une solution efficace, rapide et reproductible dans le traitement chirurgical des fractures stade 2 et 3.
- En cas de complexité de la fracture, la réduction anatomique des tubérosités et de la bascule céphalique est le seul garant d'une bonne évolution.

## References

[1] Koval KJ, Gallagher MA, Marsicano JG (1997). Functional outcome after minimally displaced fractures of the proximal part of the humerus. J Bone Joint Surg Am..

[2] Fjalestad T, Stromsoe K, Blücher J, Tennoe B (2005). Fractures in the proximal humerus: functional outcome and evaluation of 70 patients treated in hospital. Arch Orthop Trauma Surg..

[3] Court-Brown CM, McQueen MM (2007). Two-part fractures and fracture dislocations. Hand Clin..

[4] Zyto K, Ahrengart L, Sperber A, Törnkvist H (1997). Treatment of displaced proximal humeral fractures in elderly patients. J Bone Joint Surg Br..

[5] Misra A, Kapur R, Maffulli N (2001). Complex proximal humeral fractures in adults-a systematic review of management. Injury..

[6] Braman J, Flatow L (2005). Decision making in difficult proximal humerus fractures: when to fix, pin, or replace. Semin Arthro..

[7] Antuna SA, Sperling JW, Cofleld RH (2008). Shoulder hemiarthroplasty for acute fractures of the proximal humerus: a minimum five-year follow-up. J Shoulder Elbow Surg..

[8] Sirveaux F, Navez G, Roche O, Molé D, Williams MD (2008). Reverse Prosthesis for Proximal Humerus Fracture, Technique and Results. Techniques in Shoulder & Elbow Surgery..

[9] Lauritzen JB, Schwarz P, Lund B, McNair P, Transbol I (1993). Changing incidence and residual lifetime risk of common osteoporosis-related fractures. Osteoporos Int..

[10] Rose SH, Melton LJ (1982). Epidemiologics features of humeral fractures. Clin Orthop..

[11] Bengner U, Johnell O (1988). Changes in the incidence of fracture of the upper end of the humerus during a 30 year period. Clin Orthop Relat Res..

[12] Barrett JA, Baron JA, Karagas MR, Beach ML (1999). Fracture risk in the US Medicare population. J Clin Epidemiol..

[13] Cuny C, Pfeffer F, Irrazi M (2002). Un nouveau clou verrouillé pour les fractures proximales de l'humérus. Rev Chir Orthop..

[14] Neer CS (1970). Displaced Proximal Humeral Fractures: Part 1: classification and evaluation. J Bone Joint Surg Am.

[15] Hawkins RJ, Bell RH, Gun L (1986). The tree-part fractures of the proximal part of the humerus. J Bone Joint Surg Am..

[16] Mestdagh H, Vigier P, Bocquet F, Butruille Y, Letendard J (1986). Résultats à long terme du traitement des fractures-luxations de l'extrémité supérieure de l'humérus. Rev Chir Orthop..

[17] Koike Y, Komatsuda T, Sato K (2008). Internal fixation of proximal humeral fractures with a Polarus humeral nail. J Orthopaed Traumatol..

[18] Burton DJ, Watters AT (2006). Management of proximal humeral fractures. Current Orthopaedics..

[19] Boughebri O, Havet E, Sanguina M, Daumas L, Jacob p, Zerkly B, Heissler P (2007). Traitement des fractures de l'extrémité proximale de l'humérus par clou Télégraph. Rev Chir Orthop..

[20] Cuny C, Pfeffer F, Irrazi M, Chammas M, Empereur F, Berrichi A, Metais P, Beau P (2002). Un nouveau clou verrouillé pour les fractures proximales de l'humérus. Rev Chir Orthop..

[21] Cuny C, Scarlat M, Irrazi M, Beau P, Wenger V, Ionescu N, Berrichi A (2008). The Telegraph nail for proximal humeral fractures: a prospective four -year study. JShoulder Elbow Surg..

[22] Cuny C, Darbelley L, Touchard O, Irrazi M, Beau P, Berrichi A (2003). Fractures à quatre fragments de l'humérus proximal traitées par enclouage léger à vis autostables: à propos de 31 cas. Rev Chir Orthop..

[23] Gerber C, Hersche O, Berberat C (1998). The clinical relevance of posttraumatic avascular necrosis of the humeral head. JShoulder Elbow Surg..

